# The clinical utility of polygenic risk scores in genomic medicine practices: a systematic review

**DOI:** 10.1007/s00439-022-02452-x

**Published:** 2022-04-30

**Authors:** Judit Kumuthini, Brittany Zick, Angeliki Balasopoulou, Constantina Chalikiopoulou, Collet Dandara, Ghada El-Kamah, Laura Findley, Theodora Katsila, Rongling Li, Ebner Bon Maceda, Henrietta Monye, Gabriel Rada, Meow-Keong Thong, Thilina Wanigasekera, Hannah Kennel, Veeramani Marimuthu, the G2MC Evidence investigators, Marc S. Williams, Fahd Al-Mulla, Marc Abramowicz

**Affiliations:** 1 South African National Bioinformatics Institute (SANBI), University of Western Cape, Cape Town, South Africa; 2 Global Genomic Medicine Collaborative, Durham, NC USA; 3 Institute of Chemical Biology, National Hellenic Research Foundation, 11635 Athens, Greece; 4 Division of Human Genetics, Department of Pathology & Institute of Infectious Diseases and Molecular Medicine, Faculty of Health Sciences, University of Cape Town, Cape Town, South Africa; 5 Human Genetics and Genome Research Institute, National Research Centre, Cairo, Egypt; 6 National Human Genome Research Institute, National Institutes of Health, Bethesda, MD USA; 7 Center for Human Genetics Services, Institute of Human Genetics, National Institutes of Health, University of the Philippines Manila, Manila, Philippines; 8 Department of Ophthalmology, University College Hospital, Ibadan, Nigeria; 9 Epistemonikos Foundation, Santiago, Chile; 10 Genetic and Metabolism Unit, Department of Paediatrics, Faculty of Medicine, University of Malaya, Kuala Lumpur, Malaysia; 11 Ministry of Health of Sri Lanka, Colombo, Sri Lanka; 12 Global Genomic Medicine Collaborative, Durham, NC USA; 13 Department of Genetics and Bioinformatics, Dasman Diabetes Institute, P.O.Box 1180, 15462 Dasman, Kuwait; 14 Genomic Medicine Institute, Geisinger, Danville, PA 17822 USA; 15 Department of Genetics and Bioinformatics, Dasman Diabetes Institute, Kuwait City, Kuwait; 16 Department of Genetic Medicine and Development, Faculty of Medicine, Université de Genève, Geneva, Switzerland

## Abstract

Genomic medicine aims to improve health using the individual genomic data of people to inform care. While clinical utility of genomic medicine in many monogenic, Mendelian disorders is amply demonstrated, clinical utility is less evident in polygenic traits, e.g., coronary artery disease or breast cancer. Polygenic risk scores (PRS) are subsets of individual genotypes designed to capture heritability of common traits, and hence to allow the stratification of risk of the trait in a population. We systematically reviewed the PubMed database for unequivocal evidence of clinical utility of polygenic risk scores, using stringent inclusion and exclusion criteria. While we identified studies demonstrating clinical validity in conditions where medical intervention based on a PRS is likely to benefit patient outcome, we did not identify a single study demonstrating unequivocally such a benefit, i.e. clinical utility. We conclude that while the routine use of PRSs hold great promise, translational research is still needed before they should enter mainstream clinical practice.

**Supplementary Information:**

The online version contains supplementary material available at 10.1007/s00439-022-02452-x.

## Introduction

Genomic medicine aims to improve health using an individual’s genomic information, e.g. a SNP genotype or DNA sequence, to inform care. Genomic medicine is defined by the National Human Genome Research Institute as a rapidly growing field involving the application of genomic information in clinical care (NHGRI website 2021). While many successful examples of genomic medicine involve implementation of programs to identify and manage monogenic disease, i.e. disease with Mendelian inheritance, it is not clear to what extent genomic medicine is being successful regarding disease with complex inheritance. Examples of such diseases are coronary artery disease, type 2 diabetes, and cancer. Complexity in such genetic traits with intricate inheritance is twofold. Firstly, the role of the environment in disease expression is usually significant, decreasing the contribution of the genome typically to around 50% (Polderman et al. 2015), which ultimately limits the predictability of the trait based on genome analysis alone. Secondly, many complex traits result from the interaction of several independent loci. Thus, complex traits can be seen as polygenic predispositions from multiple quantitative trait loci, that eventually produced disease under the influence of a particular environmental or epigenetic modifier. Progress in Genome-wide association studies (GWAS) have identified many such quantitative trait loci, but more remain to be discovered. GWAS are research methods utilized to detect the association between genetic variants and traits in population samples. These studies are designed to improve the understanding of the biology of disease, under the assumption that a better understanding will lead to better prevention or better treatment. The GWAS data generated from human studies proved to be useful in creating genetic predictors for complex traits by estimating the effect size at multiple loci in a discovery sample and using those estimated SNP effects in independent samples to generate a polygenic risk score (PRS). (Visscher et al. 2017). Different PRS methods model the polygenic associations to the phenotype or traits in different ways, and often make distinct or similar modeling assumptions on the effect size distribution. These assumptions can frequently help in the understanding of the performance of PRS methods across phenotype with distinct genetic architectures. While most PRSs have been developed from defined populations, e.g., FinnGen (Mars et al. 2020), they seem at least partially valid in other populations as well (Dikilitas et al. 2020; Ho et al. 2020). Nonetheless, genetically diverse studies are mandatory to cover different world populations to ensure equitable clinical utilization of PRSs (Martin et al. 2019).

The generation of PRSs is a relatively novel statistical method that associates the collectively weighted risk alleles at many of a person’s SNP loci to a trait. Thus, PRS is a quantifiable genetic risk score, determined by the cumulative impact of genome-wide variants, aimed to improve risk prediction for common chronic diseases such as coronary artery disease. (Khera et al. 2018).

With empirical improvements over time, PRSs have been widely applied in many research studies of common chronic diseases, confirming their ability to predict disease risk or status, i.e., demonstrating clinical validity. According to the CDC ACCE model (Analytical validity, Clinical validity, Clinical utility and Ethical, legal & Social implication) refers to the power of a test to predict a particular clinical outcome or phenotype (CDC website: https://www.cdc.gov/genomics/gtesting/acce/index.htm). Clinical utility, on the other hand, is focused on the effect of the use of a given test on patient health outcomes. (Haddow and Palomaki 2003). The ability to predict disease occurrence using a PRS should eventually translate into clinical utility if these are to be implemented in clinical care. PRSs for some diseases were able to identify subgroups of patients with high relative risks, and absolute relative risks that approach risk values conveyed by highly penetrant, single-gene mutations (Khera et al. 2018), considered clinically actionable. PRSs have been shown to provide additional risk stratification when combined with single-gene mutation testing for monogenic disorders with incomplete penetrance, e.g., hereditary breast and ovarian cancer or familial hypercholesterolemia (Fahed et al. 2020). Stratifying the risks of common cancers, or of coronary artery disease, should presumably help tailor screening intervals, or drug regimens, respectively, and hence mitigate the disease associated with the genetic risks. Evidence for such clinical utility has however been lagging. This is because the complexity of genetic architecture and multidimensionality of genetic and environmental contributions to disease phenotypes continue to pose significant challenges for the clinical utility as well as broad-scale use of PRSs.

The aim of this study was to perform a systematic review of the existing evidence of clinical utility of PRS for genomic medicine applications. We focused our search on studies which demonstrated a benefit on patient clinical outcome, be it process outcome, intermediate outcome, or health outcome. In the case of hypercholesterolemia-related vascular disease, these would correspond for example to: the effective adoption of a healthy diet; a lowered blood LDL-cholesterol; and a decreased rate of myocardial infarction, respectively.

## Methods

Preferred Reporting Items for Systematic Reviews and Meta-Analysis (PRISMA) guidelines were followed (Moher et al. 2009; Parums 2021). This review was not registered in a systematic review register. Search terms were selected by convening a group of expert researchers in the field to consider PubMed’s Medical Subject Headings (MeSH) terms associated with the inclusion criteria. Searches combined sets of terms for genomic medicine, clinical utility and multifactorial inheritance. Terms were iteratively refined through review of results for relevance by the expert panel until consensus on these terms was reached. The full search strategy is shown in Table 1. The literature search was conducted in PubMed on articles published on or before December 16, 2020. The search was limited to publications in English. Articles retrieved were downloaded into an Excel spreadsheet, where duplicates were removed. Title and abstract screening were undertaken by five pairs of researchers (JK, LF, AB, TW, TK, CC, MT, EM, GE, HM) who each worked independently on 20% of the retrieved articles to determine if the article should be included or excluded. An identical search was conducted in PubMed on November 03, 2021 by JK, MA, FA, to ensure any recent publications were included in this review.

**Table 1:**
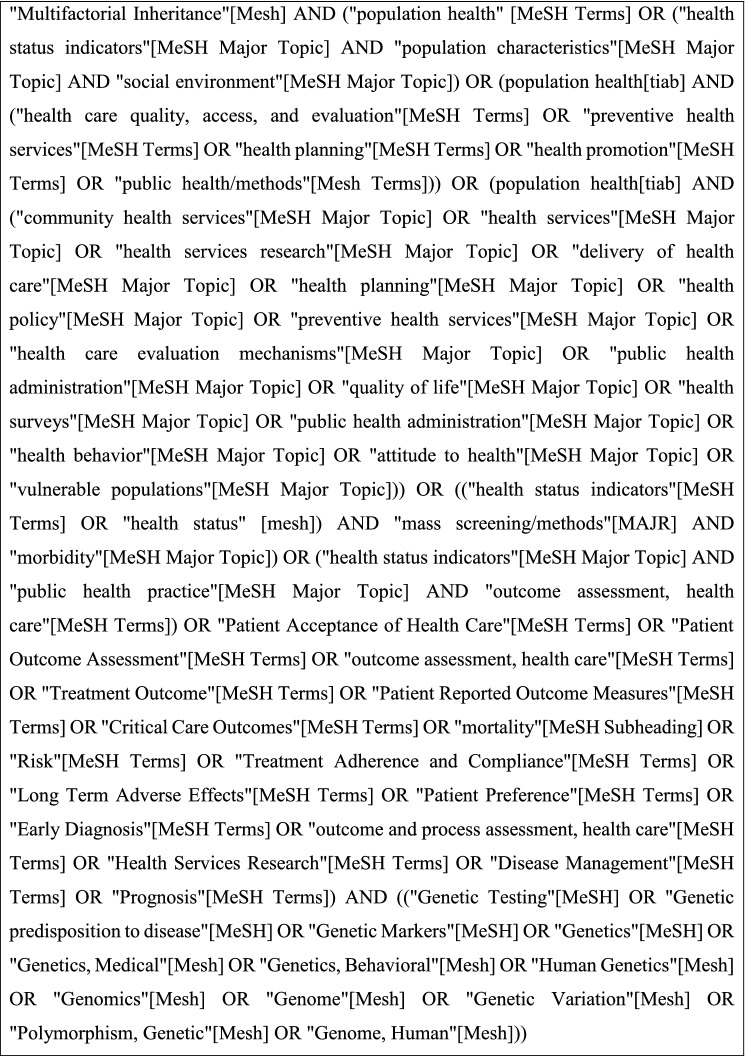
Search terms

**Fig. 1:**
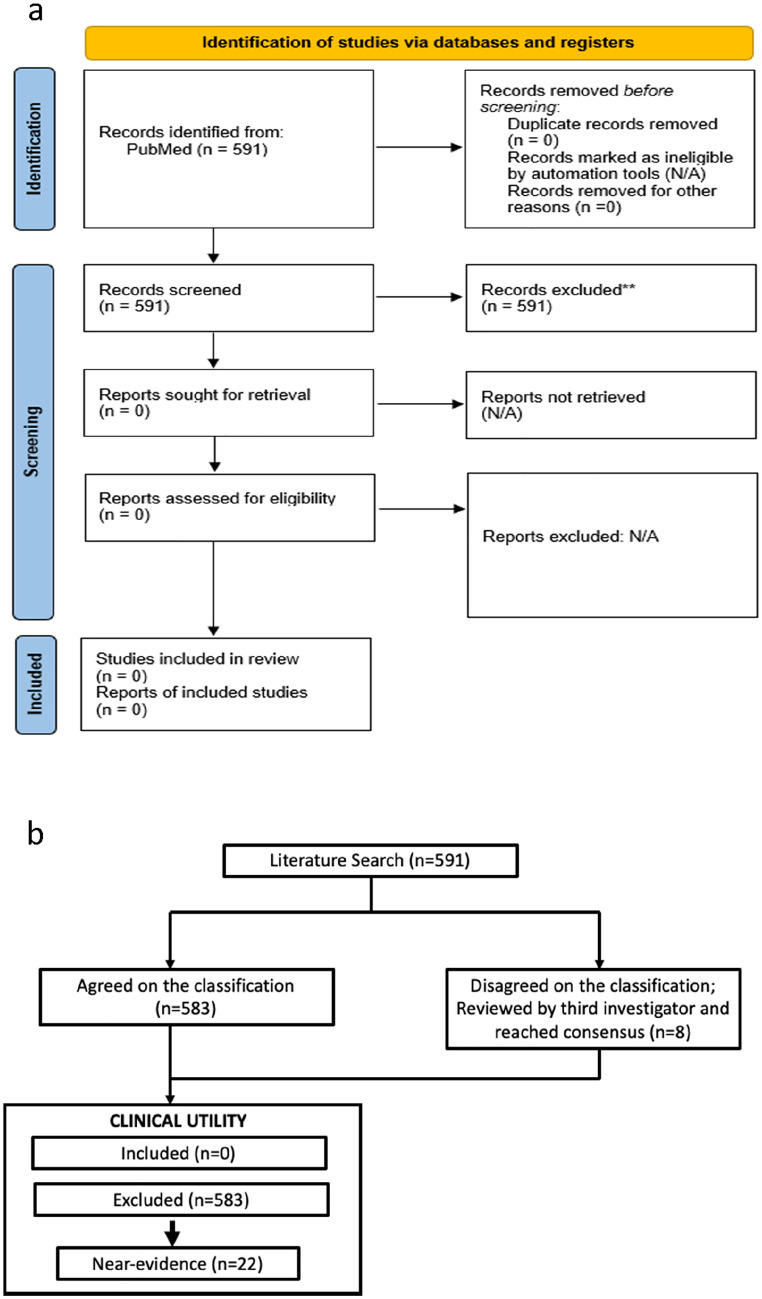
Study design and results. **a** Overview of the literature review process. **b** Outcome of the systematic review process of peer-reviewed literature following PRISMA guidelines

Included articles presented evidence of clinical utility of genomic medicine for conditions stemming from a polygenic risk where PRSs were used to inform intervention. Articles were excluded if the research findings were specific to monogenic disease, pharmacogenomics, microbial/metagenomics, expression profiling, somatic genome or methodology only. Articles were also excluded if they did not contain genomic data or health outcomes or if the articles were reviews, or association/observation studies. Studies that fulfilled all inclusion criteria and passed all exclusion criteria but failed to unequivocally demonstrate an effect on patient health outcome were additionally assigned the label of “near evidence.” These articles included evidence of clinical validity and were suggestive of utility but lacked clinical outcome data. Upon completion of the independent review of the articles, researchers compared screening results and resolved discordance with a third researcher. The final set of articles were agreed on by the entire research team (Fig. 1a). Inclusion/exclusion criteria are shown in Table 2 and additionally assigned a label of “near evidence” (see Table S1).

**Table 2:** Exclusion criteria

● Monogenic disease ● NOT genomic data ● NOT clinical utility, no health outcome ● Pharmacogenomics ● Cancer studies—tumor profiling ● Microbial/metagenomics ● Expression profiling ● Association or observation study ● Methodology only ● Review
● Other: meta-analysis, case report, interview, educational article

## Results

The initial PubMed query run on December 16, 2020, retrieved 530 articles. Ten investigators manually curated 105 articles each, and each article was curated in duplicate. Duplicate curations agreed on classifying 522 and disagreed on 8. These 8 articles were reviewed by a third investigator and discussed with the two initial investigators to reach a final consensus. This process was repeated on a second PubMed query run on November 03, 2021, and retrieved 61 additional items. The same paired review process was followed, and the results were 100% in agreement with having no clinical utility (Fig. 1b).

No study was found that showed unequivocal demonstration of clinical utility of any PRS. The study team therefore excluded the studies that were categorized as “Near-Evidence” in analysis (Fig. 1b).

22/591 studies showed robust evidence of clinical validity, i.e. some PRSs accurately stratified individual disease susceptibility, e.g., breast cancer (Mavaddat et al. 2019) or atrial fibrillation (Mars 2020). One example was PRS for breast cancer, where enhanced screens (mammograms) were likely, but not proven, to benefit women with highest risk scores, by analogy with *BRCA1&2* (Kramer et al. 2020).

## Discussion

We followed PRISMA guidelines to systematically review the PubMed database for published evidence of clinical utility of using a PRS for improving patient health, and manually curated the retrieved items to systematically remove studies dealing with monogenic disease, pharmacogenomics, microbial/metagenomics, expression profiling, somatic genome or methodology only. Our screen did not identify a single study demonstrating evidence of clinical utility of a PRS, as of November 3rd, 2021. This suggests that PRSs are not ready to be implemented in the clinic without further research. We did find studies that demonstrated clinical validity of PRSs in clinical conditions where medical action based on the PRS is likely to produce a benefit to patient outcome, which we referred to as ‘near evidence’ of clinical utility. For example, Kramer et al. (2020) demonstrated that a PRS was clinically valid in women with breast cancer for stratifying the risk of contralateral breast cancer, and concluded that this PRS “can be incorporated into contralateral breast cancer risk prediction models to help improve stratification and optimize surveillance and treatment strategies”. However, further studies are needed to demonstrate the utility of PRS prospectively does improve morbidity and mortality.

Our study has several limitations. We screened the PubMed database only, because it is a large repository of regularly updated peer-reviewed medical articles that captures a very large portion of medical knowledge. We chose not to include “gray” literature in our search, to minimize the chance of reporting false positive results, i.e. PRSs with no demonstrated clinical utility.

Our search of the PubMed literature was designed to minimize alpha and beta-type errors, but was not perfect. An additional, non-systematic approach identified a study that did demonstrate clinical utility on an intermediate outcome (blood level of LDL-cholesterol), albeit not on health outcome per se (myocardial infarction) (Kullo et al. 2016). It remains possible that more studies were not included despite demonstrating evidence of clinical utility, but we consider this possibility unlikely because our manual curation of the PubMed screen was performed in duplicate and reviewed by a third expert in case of disagreement.

We believe the main limitation of our review arises from the pragmatic decisions made to cope with the massive volume of literature on this topic. We searched for a single, albeit comprehensive source but did not attempt to identify unpublished studies or gray literature, and we did not intend to generate pooled estimates. With the huge number of studies being produced and published every year, it is very difficult to synthesize the evidence with traditional systematic review methods. One alternative is to apply artificial intelligence and other technologies that automate or semi-automate the different steps of the systematic review process. However, it is important to be very specific about what artificial intelligence can provide and where its use might be inappropriate. Exemplary reviews combining artificial intelligence with rigorous systematic review methods have been produced in the context of COVID-19 (Boutron et al. 2020; Pierre et al. 2021; Siemieniuk et al. 2020).

In spite of the current absence of unequivocal evidence of clinical utility using stringent criteria, the routine use of PRSs hold great promise. They can be assessed at low cost (< $30) at any point in time. Further research should now aim at comparing the current standard of care with and without the use of PRSs in cohorts of patients with complex traits to demonstrate a benefit for patient health. Randomized controlled studies are the best approach. An example of such a study design might consist of implementing the use of a PRS in making decisions regarding apparently benign breast tumors identified on routine mammogram screens in asymptomatic women, versus non-use of PRS in a random control cohort, and assess outcome in terms of invasive breast cancer after a defined interval, e.g., one year. It should be noted however that randomized controlled studies will be much harder to achieve where the relevant health outcomes take many years or decades to manifest (e.g. myocardial infarction). Furthermore, they must cover the diverse ethnicities of patients. Hence, some PRSs are likely to be implemented empirically for clinical decision-making, by analogy with monogenic testing, in extreme strata (both tails) of polygenic risk, e.g., for shortening screening intervals in women with high breast cancer PRS, with a posteriori*,* retrospective evaluation of clinical outcome. Another line of research should continue to improve the technical ability of PRSs to capture trait heritability. It is essential that future studies of PRSs for both clinical validity and utility engage diverse populations to improve their relevance to all population groups and avoid exacerbation of health inequities.

In conclusion, although our search could not identify published evidence of unequivocal clinical utility of a PRS, we found numerous examples of near evidence of clinical utility and ample demonstration of clinical validity. As PRS continue to improve in their ability to capture heritability of polygenic traits, we can expect demonstration of clinical utility by appropriate clinical trials in the coming years in a variety of disorders like coronary artery disease or common cancers, ushering a new era of genomic medicine.

## Supplementary Information

Below is the link to the electronic supplementary material.

Supplementary file1 (DOCX 22 KB)
