# Supplementary material for: The clinical utility of polygenic risk scores in genomic medicine practices: a systematic review
Source: Hum Genet. 2022 Apr 30;141(11):1697–704. doi: 10.1007/s00439-022-02452-x (PMC9055005; doi:10.1007/s00439-022-02452-x)
Supplement: Supplementary file 1 — Supplementary file1 (DOCX 22 KB) [file 439_2022_2452_MOESM1_ESM.docx]

**Supplementary table S1**: features of 22 publications with near evidence of clinical utility

| # | Title | Authors | Journal/ Publication year | Disease type | Sample number | Ancestry |
| --- | --- | --- | --- | --- | --- | --- |
| 1 | Genome-wide polygenic scores for common diseases identify individuals with risk equivalent to monogenic mutations | Khera AV, Chaffin M, Aragam KG, et al. | Nat Genet, 2018 | Coronary artery disease; Atrial fibrillation; Type 2 diabetes; inflammatory bowel disease; Breast cancer | 288,278 | Caucasian |
| 2 | Polygenic Risk Scores for Prediction of Breast Cancer and Breast Cancer Subtypes | Mavaddat N, Michailidou K. et al. | Am J Hum Genet, 2019 | Breast Cancer | 94,075 | Caucasian |
| 3 | Identification of risk loci and a polygenic risk score for lung cancer: a large-scale prospective cohort study in Chinese populations | Dai J, Lv J, Zhu M. et al | Lancet Respir Med, 2019 | Lung Cancer, non-small cell | 27,120 | Chinese |
| 4 | Evaluation of Polygenic Risk Scores for Breast and Ovarian Cancer Risk Prediction in BRCA1 and BRCA2 Mutation Carriers | Kuchenbaecker KB, McGuffog L, et al. | J Natl Cancer Inst, 2017 | Breast and Ovarian cancer | 15,252 female BRCA1 and 8211 BRCA2 carriers | - |
| 5 | Genomic Risk Prediction of Coronary Artery Disease in 480,000 Adults: Implications for Primary Prevention | Inouye M, Abraham G, Nelson CP, et al. | J Am Coll Cardiol, 2018 | Coronary Artery Disease | 480,000 | Caucasian |
| 6 | Common polygenic variation enhances risk prediction for Alzheimer's disease | Escott-Price V, Sims R, Bannister C, Harold D, et al. | Brain, 2015 | Alzheimer's disease | 17,008 | Caucasian |
| 7 | Penetrance and Pleiotropy of Polygenic Risk Scores for Schizophrenia in 106,160 Patients Across Four Health Care Systems | Zheutlin AB, Dennis J, Karlsson Linnér R, et al. | Am J Psychiatry, 2019 | Schizophrenia | 106,160 | Caucasian |
| 8 | Polygenic risk score analysis of pathologically confirmed Alzheimer disease | Escott-Price V, Myers AJ, Huentelman M, Hardy J. | Ann Neurol, 2017 | Alzheimer disease | 1,011 | Caucasian |
| 9 | Genome-wide Modeling of Polygenic Risk Score in Colorectal Cancer Risk | Thomas M, Sakoda LC, Hoffmeister M, et al. | Am J Hum Genet, 2020 | Colorectal Cancer | 55,105 | Caucasian |
| 10 | Polygenic Risk Score Contribution to Psychosis Prediction in a Target Population of Persons at Clinical High Risk | Perkins DO, Olde Loohuis L, Barbee J, Ford J, | Am J Psychiatry, 2020 | psychosis | 764 | Caucasian |
| 11 | Predictive Accuracy of a Polygenic Risk Score-Enhanced Prediction Model vs a Clinical Risk Score for Coronary Artery Disease | Elliott J, Bodinier B, Bond TA, Chadeau-Hyam M, et al. | JAMA, 2020 | coronary artery disease | 352,660 | Caucasian |
| 12 | Breast Cancer Polygenic Risk Score and Contralateral Breast Cancer Risk | Kramer I, Hooning MJ, Mavaddat N, Hauptmann M, et al. | Am J Hum Genet, 2020 | Breast cancer, contralateral | 56,068 | Caucasian |
| 13 | Polygenic background modifies penetrance of monogenic variants for tier 1 genomic conditions | Fahed AC, Wang M, Homburger JR, Patel AP, et al. | Nat Commun, 2020 | familial hypercholesterolemia, hereditary breast and ovarian cancer, and Lynch syndrome | 80,928 | Caucasian |
| 14 | A Weighted Genetic Risk Score Based on Four APOE-Independent Alzheimer's Disease Risk Loci May Supplement APOE E4 for Better Disease Prediction | Zhang C, Hu R, Zhang G, Zhe Y, Hu B, He J, Wang Z, Qi X. | J Mol Neurosci, 2019 | Alzheimer's Disease | 499 | Chinese |
| 15 | A Genome-Wide Test of the Differential Susceptibility Hypothesis Reveals a Genetic Predictor of Differential Response to Psychological Treatments for Child Anxiety Disorders | Keers R, Coleman JR, Lester KJ, Roberts S, Breen G, et al. | Psychother Psychosom, 2016 | Anxiety disorders | 1,026 monozygotic twin pairs | Caucasian |
| 16 | The impact of a panel of 18 SNPs on breast cancer risk in women attending a UK familial screening clinic: a case-control study | Evans DG, Brentnall A, Byers H, Harkness E, Stavrinos P, Howell A; FH-risk study Group, Newman WG, Cuzick J. | J Med Genet, 2017 | Breast Cancer | 364 | Caucasian |
| 17 | Development of a polygenic risk score to improve screening for fracture risk: A genetic risk prediction study | Forgetta V, Keller-Baruch J, Forest M, Durand A. et al. | PLoS Med, 2020 | Fracture Risk | 346,784 | Caucasian |
| 18 | Combined Utility of 25 Disease and Risk Factor Polygenic Risk Scores for Stratifying Risk of All-Cause Mortality | Meisner A, Kundu P, Zhang YD, Lan LV, et al. | Am J Hum Genet, 2020 | Mortality, overall | >500,000 | Caucasian |
| 19 | Breast cancer risk prediction using a polygenic risk score in the familial setting: a prospective study from the Breast Cancer Family Registry and kConFab | Li H, Feng B, Miron A, Chen X, Beesley J, et al. | Genet Med, 2017 | Breast cancer | 4,365 | Caucasian |
| 20 | European polygenic risk score for prediction of breast cancer shows similar performance in Asian women | Ho WK, Tan MM, Mavaddat N, et al. | Nat Commun, 2020 | Breast Cancer | 17,262 | Chinese |
| 21 | Pan-cancer analysis demonstrates that integrating polygenic risk scores with modifiable risk factors improves risk prediction | Kachuri L, Graff RE, Smith-Byrne K, Meyers TJ, Rashkin SR, Ziv E, Witte JS, Johansson M. | Nat Commun, 2020 | Cancer, 16 types | 413,753 | Caucasian |
| 22 | Assessing thyroid cancer risk using polygenic risk scores | Liyanarachchi S, Gudmundsson J, Ferkingstad E, et al. | Proc Natl Acad Sci U S A, 2020 | Thyroid cancer | 2,801 | Caucasian |
